# Neural Control Adaptation to Motor Noise Manipulation

**DOI:** 10.3389/fnhum.2016.00059

**Published:** 2016-03-01

**Authors:** Christopher J. Hasson, Olga Gelina, Garrett Woo

**Affiliations:** Neuromotor Systems Laboratory, Department of Physical Therapy, Movement and Rehabilitation Sciences, Northeastern UniversityBoston, MA, USA

**Keywords:** neural noise, motor control and learning/plasticity, antagonistic co-activation, voluntary movement, motor noise, virtual arm, signal-dependent noise, electromyography

## Abstract

Antagonistic muscular co-activation can compensate for movement variability induced by motor noise at the expense of increased energetic costs. Greater antagonistic co-activation is commonly observed in older adults, which could be an adaptation to increased motor noise. The present study tested this hypothesis by manipulating motor noise in 12 young subjects while they practiced a goal-directed task using a myoelectric virtual arm, which was controlled by their biceps and triceps muscle activity. Motor noise was increased by increasing the coefficient of variation (CV) of the myoelectric signals. As hypothesized, subjects adapted by increasing antagonistic co-activation, and this was associated with reduced noise-induced performance decrements. A second hypothesis was that a virtual decrease in motor noise, achieved by smoothing the myoelectric signals, would have the opposite effect: co-activation would decrease and motor performance would improve. However, the results showed that a decrease in noise made performance worse instead of better, with no change in co-activation. Overall, these findings suggest that the nervous system adapts to virtual increases in motor noise by increasing antagonistic co-activation, and this preserves motor performance. Reducing noise may have failed to benefit performance due to characteristics of the filtering process itself, e.g., delays are introduced and muscle activity bursts are attenuated. The observed adaptations to increased noise may explain in part why older adults and many patient populations have greater antagonistic co-activation, which could represent an adaptation to increased motor noise, along with a desire for increased joint stability.

## Introduction

It could be argued that part of what makes the study of human movement interesting is its variability. This variability arises, in part, from noise within the nervous system (Faisal et al., 2008). This noise is present in motor commands and increases with signal magnitude, i.e., motor noise is signal-dependent. Motor noise has important implications for human movement control—the amplitude of motor commands should be kept small to minimize the variability of motor actions (Harris and Wolpert, 1998; Todorov, 2004). However, even with small motor commands, noise impacts may be severe if there is a high demand for movement accuracy. Fortunately, the human neuromotor control system has at least one strategy to increase accuracy independent of motor command magnitude: antagonistic co-activation. Experimental studies have demonstrated that increasing task accuracy demands elicits increases in antagonistic co-activation (Laursen et al., 1998; Osu and Gomi, 1999; Gribble et al., 2003). Antagonistic co-activation increases limb impedance, which can reduce kinematic variability by acting as a mechanical filter (Selen et al., 2005, 2009).

Although there have been many investigations on the consequences of motor noise for human movement control, less is known about how the nervous system adapts to changes in motor noise, which may not be a fixed quantity. Motor noise may increase with aging (Holloszy and Larsson, 1995; Laidlaw et al., 2000; Kallio et al., 2012) and disease states, such as with Parkinson’s disease (Winterer et al., 2004). This could explain why many older adults and patient populations have increased levels of co-activation, as documented by others (Izquierdo et al., 1999; Macaluso et al., 2002; Hortobágyi and Devita, 2006), which may be a strategy to suppress variability. However, this hypothesis remains untested, in part due to the difficulty of manipulating motor noise in living humans. Direct manipulation of motor noise could be achieved by injecting noisy electrical currents into the motor axons of humans, but this poses ethical challenges.

An alternative to an invasive approach is to use a myoelectrically controlled virtual arm, in which the motor command, represented by myopotentials recorded through surface electromyography, can be intercepted and manipulated, prior to serving as input to a virtual arm model. In concept, this approach is analogous to a muscle activity-driven prosthetic arm. While manipulation of motor noise in this context is artificial, it could provide insight into how the nervous system would adapt to a real increase in motor noise. Using a similar approach, de Rugy et al. (2012) increased the noisiness of motor commands as participants performed fast ramp-and-hold isometric exertions to achieve a target force magnitude in different directions. The resultant force depended on a virtual mapping of muscle activity from five muscles. When the noisiness of one muscle’s activity was (virtually) increased, participants did not change their control of the manipulated muscle, nor did the activity of synergistic and antagonistic muscles change. These results are inconsistent with the hypothesis that humans use co-activation to suppress variability, and could be because musculotendon and rigid-body dynamics were excluded from the virtual arm model and the task was highly constrained, preventing subjects from taking advantage of the impedance-modulating capabilities of antagonistic muscles (Hogan, 1984). Whether different results would be obtained using a virtual arm model with specific representations of musculotendon structures in a more ecological task setting remains an open question.

Further, how humans adapt to decreased motor noise is relatively unknown. In theory, such a decrease could be doubly-beneficial: it would decrease variability and improve movement economy by reducing the need for antagonistic co-activation. Whether it is possible to decrease motor noise stemming from basic physiological processes is unclear; however, other strategies may be used to reduce the levels of motor noise associated with motor tasks. For example, by increasing the strength of a muscle, less neural drive is needed to produce a given force, and therefore, motor noise is attenuated because the noise is signal-dependent. Chu et al. (2013) found that dystonic children can take advantage of a perceived decrease in their motor variability in a skilled motor task. However, it is unclear whether individuals with a healthy neuromotor system would benefit in the same way, and changes in antagonistic co-activation were not assessed.

Therefore, the aim of this study was to investigate how the nervous system adapts to virtual manipulations of motor noise. It was hypothesized that: (1) humans adapt to a virtual increase in motor noise by increasing antagonistic co-activation, and this reduces noise-induced performance decrements and (2) humans adapt to a virtual decrease in motor noise by decreasing antagonistic co-activation, and this improves performance. To test these hypotheses, motor noise was manipulated in 12 healthy young adults, who used a myoelectric virtual arm to perform a goal-directed task. After the subjects became proficient at the task, the noise in their muscle activity was either increased or decreased, and changes in task performance and neural adaptations assessed.

## Materials and Methods

### Overview

**Figure 1:**
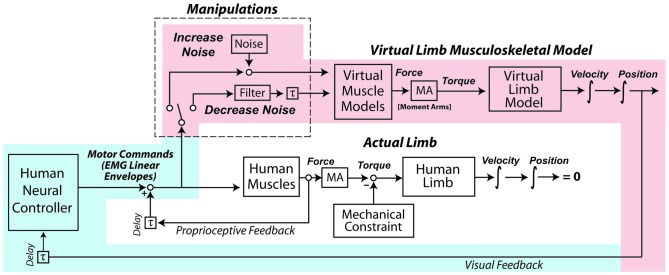
**In the experiment, subjects used their muscle activity to perform a goal-directed task with a virtual arm.** After a period of familiarization, the coefficient of variation (CV) of subjects’ motor commands (as reflected by muscle activity linear envelopes) was increased by adding signal-dependent noise, and in another condition the CV was decreased with an exponentially-weighted moving average filter. The shading highlights the control loop hypothesized to take precedence in the virtual arm task, with processes associated with human physiology shaded in light blue, and processes in the virtual world shaded in light red. Note that during the experiment the actual human arm does not move due to an external mechanical constraint, and although subjects received proprioceptive feedback about their actual arms, this feedback did not reflect the actions of the virtual muscles and arm, and therefore was not of high relevance to the task (in contrast to the visual feedback).

The experiment occurred over two days. The first day was a familiarization session so subjects could gain proficiency at controlling a myoelectric virtual arm. On the second day, subjects performed a number of isometric exertions with their real arms, from which estimates of motor noise were obtained. Subjects then performed the virtual arm task with no added noise, with increased noise, and with decreased noise. Each subject’s motor noise was increased and decreased by the same relative proportion, based on the measurements taken during the isometric exertions. A schematic detailing the virtual arm model and noise manipulations is presented in Figure 1.

### Subjects

Twelve healthy young subjects participated in the study (age = 24 ± 1.7 yrs; height = 1.69 ± 0.11 m; weight = 66.9 ± 10.9 kg [mean ± standard deviation (STD)]; 5 males; 7 females). All subjects were right-hand dominant, were healthy, and had no neurological or musculoskeletal issues that affected movement control. The study was approved by the Northeastern University Institutional Review Board, and all subjects signed an informed consent form prior to participation.

### Experimental Setup

#### Apparatus

Participants sat in a chair and faced a computer monitor. Their right shoulder was in the anatomical position (aligned with and next to the torso), the elbow was flexed 90°, and the wrist was placed in a neutral position. The forearm and wrist rested on a cushion and were strapped to a rigid aluminum frame using Velcro straps to allow isometric elbow flexion and extension actions. Subjects’ arms remained in this fixed position throughout the experiment.

#### Electromyography

A wireless electromyography system (Myon AG, Baar, Switzerland; bandwidth: 5–1000 Hz, latency: 16 ms) was used to monitor the biceps and triceps brachii. For the biceps, bipolar Ag/AgCl circular disposable electrodes (Kendall^TM^Arbo^TM^ H124SG, Covidien, UK, Commercial Ltd) were orientated parallel to the muscle fibers, and placed 1 cm laterally from the septum between the biceps muscle heads determined by palpation (Riley et al., 2008). The other set of electrodes were placed on the lateral head of the triceps, oriented parallel to the muscle fibers. The inter-electrode distance was 2.0 cm. Prior to electrode placement, the skin was shaved, rubbed with an abrasive gel (NuPrep^®^, Weaver and Company, Aurora, CO, USA), and cleaned with alcohol. After placement the electrodes were covered with elastic wrap. Amplified muscle activity was rectified and filtered using an analog fifth-order low-pass Butterworth filter (MAX280; Maxim Integrated Products, Inc., San Jose, CA, USA) with a cutoff frequency of 4 Hz (Manal et al., 2002; Hasson and Manczurowsky, 2015). These linear envelopes were sampled at 100 Hz using an analog-to-digital converter (PCI-6289; National Instruments, Austin, TX, USA).

### Musculoskeletal Model

#### Virtual Arm Model

Subjects controlled a myoelectrically driven virtual arm model (Hasson, 2014; Hasson and Manczurowsky, 2015). The virtual arm model was created in Matlab^®^ (MathWorks^®^, Natick, MA, USA). The virtual arm rotated about a hinge joint with a moment of inertia equal to 0.24 kgm^2^ (Winter, 1990), which approximated a human forearm and hand. A pair of antagonistic two-element lumped Hill-type (Hill, 1938; Zajac, 1988) muscle models produced forces to accelerate the arm. One muscle model represented the behavior of the elbow flexors and a second modeled the elbow extensors. Each muscle model contained a contractile element in series with an elastic element; the behavior of these elements was specified by a maximal isometric strength (P_0_), a length-dependent strength defined by a force-length relation (Gordon et al., 1966), a velocity-dependent strength defined by a force-velocity relation (Hill, 1938), and a series-elasticity defined by a force-extension relation (Bahler, 1967). Parameters defining these relationships were adapted from the SIMM (MusculoGraphics, Inc., Santa Rosa, CA, USA) musculoskeletal modeling software (Delp et al., 1990); see Hasson and Manczurowsky (2015) for parameter details. The lumped flexor and extensor length vs. angle relation was the average of the relations for the SIMM elbow flexor and extensor muscles, respectively. The individual muscle SIMM moment arm vs. elbow angle relations were averaged to produce relations for the lumped flexor and extensor muscle models. An elastic torque prevented the virtual limb from circling around the axis of rotation, and a frictional torque was added to mimic a limb rotating on a planar surface. Further details about the musculoskeletal model and passive torques are provided in Hasson (2014), and the equations describing the muscle model dynamics are provided in Hasson and Caldwell (2012).

### Noise Manipulations

#### Increasing Noise

**Figure 2:**
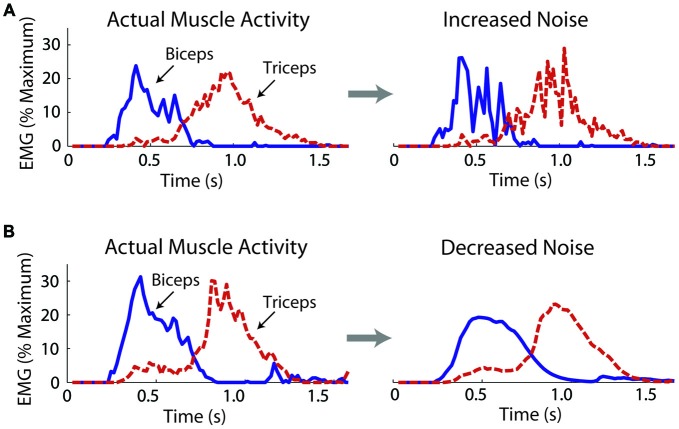
**Exemplar data from one subject showing actual and manipulated muscle activity during the virtual arm task.** Noise was increased by adding noise to the muscle excitation linear envelopes before these signals activated muscle models, which produced forces to move the virtual arm **(A)**. Noise was decreased by applying a low-pass filter **(B)**.

We operationally defined motor noise as the noise contained within the muscle linear envelopes. While this noise likely contains components due to measurement noise, such components remained constant across the experimental conditions. To increase motor noise, Gaussian distributed noise was added to the biceps and triceps muscle linear envelopes, which subjects used to control the virtual arm model. The STD (*σ*) of the added noise increased linearly with the excitation signal, i.e., the noise was multiplicative. The noise was added in a muscle- and subject-specific manner, doubling the coefficient of variation (CV) for each muscle of each subject (a 100% increase; a change in intensity of about 6 dB). A doubling of the CV was chosen based on Laidlaw et al. (2000), who showed an age-related doubling (approximately) of the CV for hand muscle motor neuron firing rates. The actual CVs of the biceps and triceps linear envelopes were calculated from a series of isometric efforts for each subject (see *“Noise Measurements”* Section). Through an iterative process noise was added to the muscle activity linear envelopes with steadily increasing values of *σ* until the CV was doubled and these *σ* values were used to increase motor noise while subjects controlled the virtual arm (see *“Virtual Arm Simulation”* Section). An example of a trial without and with increased noise is shown in Figure 2A.

#### Decreasing Noise

To reduce variability, the linear envelopes were smoothed with an exponentially weighted moving average. A moving-average filter was used because it is the fastest type of digital filter and is optimal with respect to reducing noise and retains a sharp response (Smith, 1997). The smoothing function was:

(1) $$s_{t}\;=\;\alpha x_{t}+(1-\alpha )s_{t-1}$$

where *α* is the smoothing factor, *x_t_* is the excitation vale at the current time, and *s_t-1_* is the previous smoothed excitation value. The smoothed excitation value at *t* = 0 was set equal to the unsmoothed excitation value. As for the noise increase, an iterative procedure was used to find muscle- and subject-specific values for *α*. Subjects’ isometric linear envelopes were smoothed with increasing values of *α* (increasingly more smooth) until a value was reached that decreased the CV by 30% (a change in intensity of about −3 dB). The noise decrease (30%) was chosen to be less than the noise increase (100%) because pilot work showed that the delay introduced by smoothing became significant with more extreme smoothing factors. Test signals were created at the simulation sampling rate (100 Hz) to estimate the filtering delay using *α* = 0.2 (close to the average used in the experiment). For a rectified 1 Hz sine function (amplitude = 1) the filter introduced a delay of 46 ms. For a step function, it took 43 ms for the filtered signal to reach 63.2% of the maximum signal value after the step. An example of a trial without and with decreased noise is shown in Figure 2B.

#### Virtual Arm Simulation

The biceps and triceps linear envelopes represent the excitation signals to the virtual flexor and extensor muscle models. These signals were converted from arbitrary voltages to a percentage of each subjects’ maximum isometric muscle activity (see “*Virtual Arm Calibration*” Section). To increase noise while subjects controlled the virtual arm, at each iteration of the simulation the data acquisition system sampled the most recent linear envelope values; random numbers were drawn separately for the biceps and triceps using muscle- and subject-specific *σ* values, and these numbers were added to the muscle excitation values. To decrease noise, the sampled muscle excitation values were smoothed with Equation 1, using muscle- and subject-specific *α* values. In non-manipulated conditions, the recorded linear envelopes were left untouched. The signals were then subjected to first-order excitation-activation dynamics with activation/deactivation time-constants of 11 and 68 ms, respectively (Winters and Stark, 1988), and converted to muscle forces via the lumped muscle models. These forces were multiplied by the muscle moment arms to produce torques about the virtual elbow axis of rotation. The sum of the muscular torques, including passive and frictional torques, was used to calculate the virtual limb angular acceleration using Euler’s equations of motion, followed by integration via a 4th-order Runge-Kutta algorithm (Press et al., 1990) to obtain virtual limb position and velocity. All computations and simulations were performed in MATLAB^®^. Details of the simulation flow are in Hasson (2014).

### Modeling Considerations

**Figure 3:**
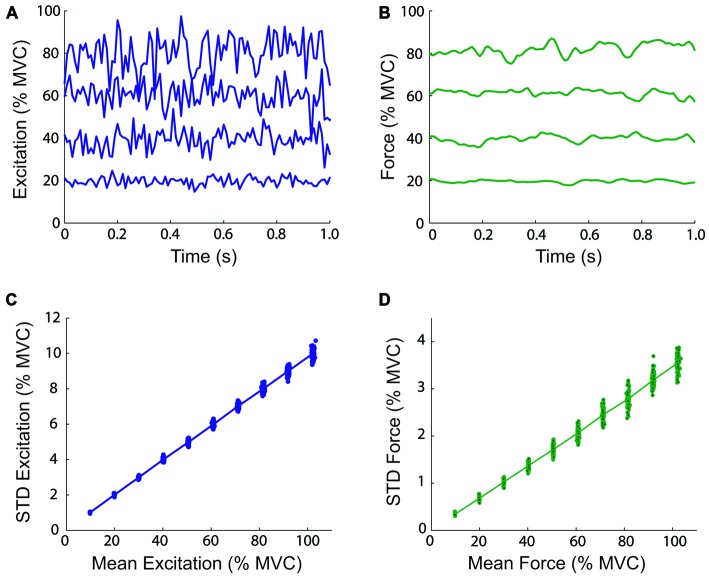
**Magnitude and variability of virtual arm neural control signals and virtual muscle forces.** The top panels show one-second examples of simulated neural excitation time-histories **(A)** and resulting virtual muscle force **(B)** at 20, 40, 60, and 80% of maximal voluntary contraction (MVC) during simulated isometric efforts of the biceps lumped muscle model. The total virtual muscle-tendon length was fixed at 0.3051 m (the length when the virtual arm was at 90° of elbow flexion). The bottom panels show the relationship between the mean and standard deviation (STD) excitation **(C)** and muscle model force **(D)** over 10 s at different mean excitation levels from 10 to 100% in 10% increments (100 simulations were performed at each excitation level).

It has been demonstrated by Selen et al. (2005) that the use of a lumped muscle model does not produce variations in force output consistent with physiological data, and that a model of the motor unit pool is needed to model signal-dependent noise. This was due to the inclusion of a length-dependency for neural activation (Endo, 1972), which introduced a low-frequency stiffness in addition to the stiffness contributed by the series elastic elements (Kistemaker et al., 2005). A model of the motor unit pool is difficult to incorporate in the real-time control of a myoelectric virtual arm without accurate on-line measurements of the activity of a person’s motor unit pool. Thus, the present study used lumped muscle models controlled by inputs from surface electromyography. By excluding the dependency of activation on muscle fiber length from the model, the linear relation between muscle activation/force and variability is preserved, at the cost of a small decrease in physiological fidelity. To verify this claim, Monte-Carlo isometric simulations were performed with the biceps lumped muscle model at mean excitation levels from 10 to 100% in 10% increments (CV = 0.2). One hundred simulations were performed at each excitation level. Examples of muscle model excitation and force time-histories, and the relation between the mean excitation/force, and the STD of the excitation/force are shown in Figure 3. These relations are linear and therefore, the CV is constant using lumped muscle models without length-dependent activation dynamics.

### Experimental Methods

#### Virtual Arm Calibration

At the start of each experiment on Day 1 and 2, electrodes were applied to the biceps and triceps muscles according to the procedure described in the “*Experimental Setup*” Section, and muscle activity was collected at rest and during maximal voluntary contractions (MVCs), allowing the strength of the virtual arm muscles to be tailored to each subject. On Day 2, this data was also used to derive target effort levels (%MVC) for the isometric efforts (see *Noise Measurements*; next section). Subjects performed three MVCs with their biceps muscle against an immovable resistance, followed by three triceps MVCs. Each MVC lasted 5 s with a 30 s rest between trials. This normally took about 5 min.

#### Noise Measurements

On Day 2, the noise in each subject’s muscle activity was measured via isometric exertions performed with the actual arm. The approach was similar to that used by Christou and Carlton (2001). Subjects performed a series of isometric muscle exertions at 5, 10, 15, 20, 25, 30, 50, and 80% of MVC. Two trials at each percentage were performed. The order of the 16 MVC percentages (8 × 2 trials each) was randomized. Trials between 5–30% lasted 15 s; trials between 50–80% lasted 10 s to lessen fatigue. Visual feedback was provided for the first 5 s of each trial, during which the instantaneous excitation magnitude was shown as a vertical bar. This visual feedback was removed for the duration of the trial; subjects were asked to maintain their muscular effort. A 30 s rest was provided after 5–30% MCV trials and a longer 60 s rest between 50–80% MVC trials to minimize fatigue. This took about 15 min for each muscle (30 min total).

#### Virtual Arm Task

**Figure 4:**
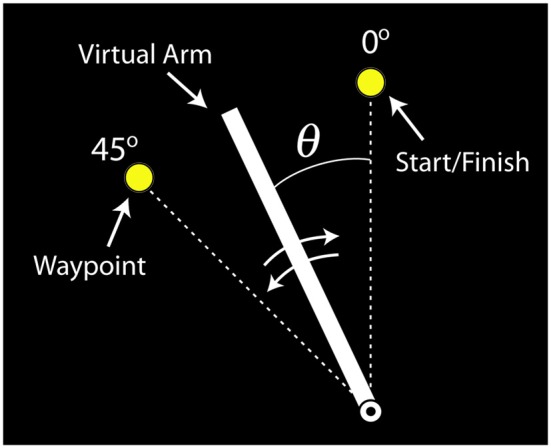
**Visual feedback of the virtual arm provided to participants (labels included for clarity).** The virtual arm started in the vertical position (0°). The task was to move the virtual arm to the waypoint (45°) and then back to the starting position, i.e., perform a back-and-forth slicing motion.

The virtual arm was drawn as a rotating line segment on a visual display (Figure 4). Subjects were instructed to use their muscle activity to perform a slice movement, which required the arm to be moved in two directions: counterclockwise to pass through a waypoint and then back to the starting location. The virtual biceps and triceps produced torques that accelerated the virtual arm counterclockwise and clockwise, respectively. If both virtual muscles produced equal, but opposite torques the arm did not accelerate (ignoring passive and frictional torque contributions). Subjects were told to move the arm back and forth as quickly as possible and to stop the arm as close to the starting target circle center as possible, and that the trial ends when the virtual arm comes to rest (<4°/s for 0.3 s). For reinforcement the target circle turned from yellow to green when the angular error was within a success threshold of ±4° from the target center, and a “ding” was sounded. After each trial the movement time was displayed, defined as the time from when the limb left the starting circle to when the limb stopped. For additional motivation, subjects’ fastest successful movement time was also displayed, and if a given trial exceeded this time the program “applauded” and the fastest time was updated. If the virtual arm did not pass the waypoint, a buzzer sounded after the trial was completed and the visual display indicated this failure.

#### Protocol

On Day 1, after calibration of the virtual arm, subjects practiced the task without manipulation for four blocks of 60 trials (240 total trials; *Initial Practice*). On Day 2, the calibration procedure was performed again, and then the *Noise Measurements* were performed. Subjects then practiced the virtual arm task for one block of 60 trials without manipulation (*More Practice*). Next, the manipulation blocks were performed in the following order: Noise Increase 1, Noise Decrease 1, Noise Increase 2, and Noise Decrease 2. The order of the manipulations was not randomized so that adaptation across the two noise increases and the two noise decreases could be examined. In each manipulation block of 60 trials, subjects first performed 15 trials without manipulation (to washout effects from a prior manipulation and serve as a comparison), and then performed 45 trials with the manipulation. Performance with each manipulation was then compared with performance on the immediately preceding pre-manipulation baseline blocks. Brief rest periods were provided between all blocks. Each block of 60 trials took about 6 min.

### Data Analysis

#### Task Performance

Subjects’ proficiency at controlling the virtual arm was quantified by absolute angular error and movement time. The error was calculated as the absolute value of the angular difference between the virtual limb’s final position and the target center. Movement time was determined as the time from when the limb left the starting circle to when it came to rest.

#### Motor Noise

The CV for each isometric effort performed during the noise measurements was calculated by dividing the average linear envelope magnitude during the last 5 s by the STD of the linear envelope. The average CV across all isometric efforts was then calculated for each subject. The CV was assessed during the time without visual feedback because otherwise, variations in the linear envelope could arise from perpetual-motor information processing (Schmidt et al., 1979; Slifkin and Newell, 2000). Of additional theoretical interest is how the CV scales with the magnitude of subjects’ control signals (i.e., linear envelopes), as this has been shown to be approximately constant experimentally (Jones et al., 2002). To determine CV scaling, for each subject a linear function was fit to the log-log relation between the average and STD of the biceps and triceps muscle activity linear envelopes. A constant CV gives a slope of 1.0. The coefficient of determination (*R*^2^) was used to assess the quality of the fits.

#### Muscular Co-Activation

Following Falconer and Winter (1985) and Kellis et al. (2003), antagonistic co-activation was calculated as:

(2) $$\text{Co-Activation}\;=\;\frac{100\;(2I_{ANT})}{I_{TOTAL}}$$

where *I_ANT_* is the area of overlap between the two muscle linear envelopes, given by:

(3) $$I_{ANT=}\{\begin{array}{cc} \int_{t_{i}}^{t_{i+1}}EMG_{BICEPS}(t)dt & \text{when}\;EMG_{BICEPS}\leq EMG_{TRICEPS}\\ \int_{t_{i}}^{t_{i+1}}EMG_{TRICEPS}(t)dt & \text{when}\;EMG_{BICEPS}>EMG_{TRICEPS} \end{array}$$

The calculation of *I_ANT_* was performed in an iterative manner, i.e., the area of overlap *I_ANT_* was determined for each consecutive chunk of time from *t_i_* and *t_i+1_*, and this procedure was iterated throughout a trial for all time points. The quantity *I_TOTAL_* is the summed area of both muscles, given by:

(4) $$I_{TOTAL}\;=\;\int_{t_{i}}^{t_{i+1}}\left[EMG_{BICEPS}+EMG_{TRICEPS}\right](t)dt$$

calculated in the same iterative fashion as for* I_ANT_*.

### Statistics

#### Non-Manipulated Trials

There were three questions of interest related to subjects’ performance on the non-manipulated trials: (1) Did subjects improve their performance on Day 1? (2) Did they retain what they learned on Day 2? (3) Did they continue to improve on Day 2? The dependent variables included the absolute angular error, movement time, and antagonist co-activation. To answer Question 1, a paired *t*-test was performed between the first and last 10 trials on Day 1. To answer Question 2, a paired *t*-test was performed between the last 10 trials on Day 1 and the first 10 trials on Day 2. Finally, to answer Question 3, which had six time points, a one-way repeated measures analysis of variance (ANOVA) was performed on the average performance on the first and last 10 trials of the initial 60-trial Day 2 adaptation period and the last 10 trials of each of the four pre-manipulation Day 2 baseline blocks. *Post hoc* comparisons were made using Bonferonni corrections.

#### Noise Measurements

Paired *t*-tests were performed to determine whether the biceps and triceps CVs, noise factors (*σ*), and smoothing factors (*α*) were different, whether the slopes of linear fits to the log-transformed linear envelope magnitude vs. STD data were different, and whether the coefficients of determination (*R*^2^) for these linear fits was different between the muscles.

#### Noise Manipulations

The statistical analysis for the noise manipulations tested the main hypotheses that: (1) humans adapt to increased motor noise by increasing antagonistic co-activation, which reduces noise-induced performance decrements and (2) humans adapt to decreased motor noise by decreasing antagonistic co-activation, which improves performance. Performance was again quantified in terms of error, movement time, and co-activation. However, these tests were performed on* relative* performance measures, i.e., performance with the noise manipulations relative to performance on the most recent non-manipulated trials. To this end, the average performance for the last 10 trials in each of the four manipulation blocks was subtracted from the average of the last 10 trials in each of the immediately preceding baseline blocks. Analyzing the relative change controlled for any continued performance improvements present on the non-manipulated trials. To determine whether the noise manipulations had an effect on performance (i.e., was the change with a manipulation different from zero?), one-sample *t*-tests were performed on the difference measures (manipulation minus preceding baseline) separately for each noise manipulation. To assess whether subjects adapted to the noise manipulations, paired *t*-tests were performed comparing the first and second manipulation difference measures (i.e., was the change in error for the first noise increase different from the change in error for the second noise increase?).

## Results

### Non-Manipulated Trials

#### Initial Skill Acquisition (Day 1)

**Figure 5:**
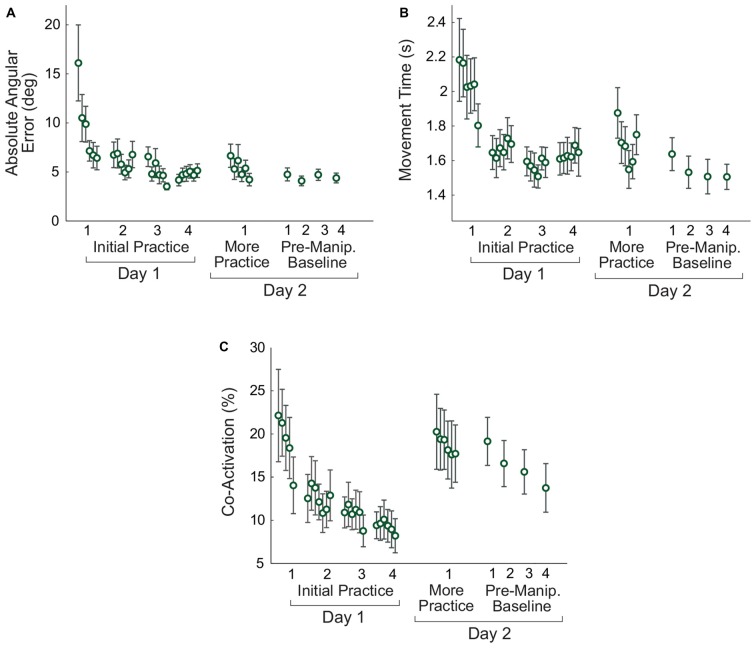
**Changes in non-manipulated virtual arm trials with practice.** Measures of task performance, the absolute angular error **(A)**, movement time **(B)**, and antagonistic co-activation **(C)** are shown. For the initial practice on Day 1 and more practice on Day 2 the data shown are averaged in non-overlapping 10-trial bins. For the pre-manipulation baseline trials, each 15-trial block is averaged to produce one data point. Error bars represent the standard error.

Subjects improved their performance on the virtual arm task on Day 1 (Figure 5). Paired *t*-tests comparing early and late practice on Day 1 showed significant decreases in error (*p* = 0.008), movement time (*p* = 0.049), and co-activation (*p* = 0.027).

#### Retention (Day 1 vs. 2)

Retention was assessed by comparing late practice on Day 1 with early practice on Day 2. Paired *t*-tests showed no differences between these two time points for error (*p* = 0.207) and movement time (*p* = 0.110), indicating that subjects retained their skill overnight. Although there was an increase in the mean co-activation between Days 1 and 2, the difference was not significant (*p* = 0.069).

#### Later Practice (Day 2)

To determine whether there were continued improvements in performance on Day 2, a repeated measures ANOVA was performed to compare six time points (the average of the first and last 10 trials of the initial 60-trial Day 2 adaptation period, and the last 10 trials of each of the four pre-manipulation Day 2 baseline blocks). Greenhouse-Geisser corrections were used due to non-sphericity. There was no significant effect of practice on error (*F*_(2.266,24.930)_ = 2.196, *p* = 0.127), but there was an effect of practice on movement time (*F*_(1.717,17.174)_ = 6.553, *p* = 0.010). *Post hoc* tests with Bonferroni corrections showed that the movement time at the end of the initial Day 2 practice period was longer than movement time for each of the last three pre-manipulation baseline blocks (*p* < 0.019). However, there were no differences between each of the four pre-manipulation blocks (*p* > 0.133). There was no significant effect of practice on Day 2 for co-activation (*F*_(2.075,22.824)_ = 2.292, *p* = 0.122).

### Noise Measurements

**Figure 6:**
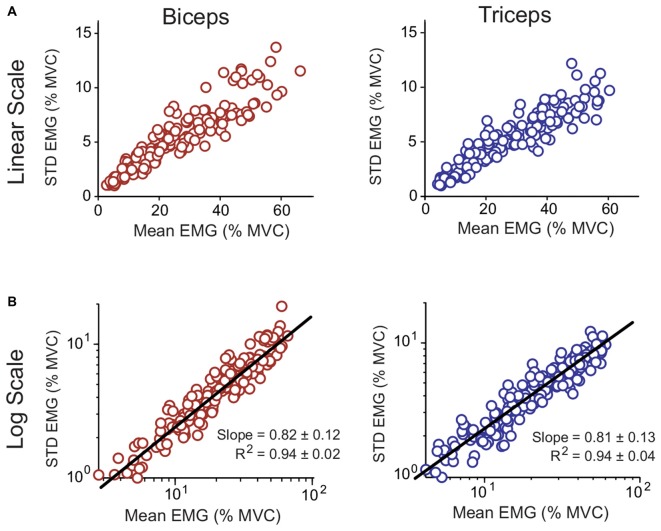
**Relation between the mean and STD of muscle linear envelopes recorded with surface electromyography (EMG) for the biceps and triceps.** Data for all subjects and all isometric efforts are shown. The data are shown both on decimal (**A**; top row) and logarithmic (**B**; bottom row) scales. A line of best fit to the pooled data is shown for the logarithmically scaled data. The *R*^2^ and slope values reported in **(B)** are for individual subject fits (mean ± STD).

The CV, which characterized each subject’s signal-dependent noise, was calculated for each biceps and triceps isometric effort by dividing the average magnitude of the muscle activity linear envelopes by the STD. The resulting data are shown in Figure 6A (top row). On average the CV was close to 0.2 for both muscles (biceps: 0.211 ± 0.026; triceps: 0.190 ± 0.021; mean ± STD). A paired *t*-test showed that the CV for the biceps was larger than the triceps (*p* = 0.004). The relation between the magnitude and STD of the muscle activity linear envelopes is shown for all subjects in Figure 6A. Of theoretical interest is the consistency of the CV across different effort levels, i.e., does the muscle activity variability scale linearly with the magnitude? This question is best addressed by taking the logarithms of the magnitude and STD; in log-log space, if the underlying process has a linear scaling then the slope will be equal to one (Jones et al., 2002). This transformed data is shown for all subjects in Figure 6B (bottom row) with a line-of-best-fit to the pooled data. The linear fits to the individual subject data had a high coefficient of determination (*R*^2^), averaging 0.94 for both muscles (no difference between muscles; *p* = 0.777), and the slopes averaged between 0.81–0.82 for both muscles (no difference between muscles; *p* = 0.755).

### Noise Manipulations

After each subject’s noise was measured on Day 2, subjects performed the virtual arm task under non-manipulated conditions, as well as with increased and decreased signal-dependent noise. The noise was manipulated according to muscle- and subject-specific noise factors. For the noise increase: biceps *σ* = 0.39 ± 0.04; triceps *σ* = 0.35 ± 0.04 (mean ± between subjects STD). For the noise decrease: biceps *α* = 0.19 ± 0.05; triceps *α* = 0.21 ± 0.04.

#### Effects of Increased Motor Noise (Hypothesis 1)

There were no statistically significant differences between the last four baseline blocks for the average error, movement time, and co-activation, suggesting a relative plateau in performance (see the results for “*Later Practice*” in preceding section). Nevertheless, there were some visible changes in antagonistic co-activation over these blocks (Figure 5C). To minimize the effects of any such trends, each subject’s average error, movement time, and co-activation on the noise manipulation trials was subtracted from their values on the immediately preceding pre-manipulation baseline trials.

**Figure 7:**
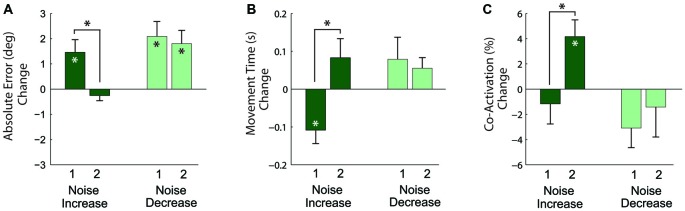
**Changes in task performance, quantified by the absolute angular error (A), movement time (B), and antagonistic co-activation (C) with increased (dark green bars) and decreased (light green bars) motor noise.** Each bar represents the change relative to a preceding block of non-manipulated trials. Measures significantly different from zero denoted by (*), measures that differ across manipulation blocks 1 and 2 denoted by connecting lines with (*) above. Error bars represent the standard error.

The results of the analysis of these relative changes in performance (Figure 7) showed that in response to the first noise increase, absolute angular error increased (*p* = 0.019) and movement time decreased (*p* = 0.013), but co-activation did not change (*p* = 0.482). For the second noise increase, absolute angular error and movement time remained the same (*p* = 0.297 and *p* = 0.153, respectively), but co-activation increased (*p* = 0.014). To assess adaptation across practice, the change between the first and second noise increase was compared. There was a decrease in error (*p* = 0.015), an increase in movement time (*p* = 0.009), and an increase in co-activation (*p* = 0.012) from the first to second noise increase blocks. Note that the increase in co-activation is opposite to the decreasing trend seen in co-activation across the pre-manipulation baseline blocks (Figure 5C); therefore, the co-activation increase is likely not an artifact of a more general practice trend.

#### Effects of Decreased Motor Noise (Hypothesis 2)

In response to the first noise decrease (Figure 7), the absolute angular error increased (*p* = 0.005), but movement time and co-activation did not change (*p* = 0.228 and *p* = 0.074). A similar pattern was observed in response to the second noise decrease: absolute angular error increased (*p* = 0.006), but movement time and co-activation remained the same (*p* = 0.077 and *p* = 0.563, respectively). To again assess adaptation across practice, the changes between the first and second noise decreases were compared. There were no significant differences for error, movement time, and co-activation (*p* = 0.660, *p* = 0.643, and *p* = 0.579, respectively) between the first and second noise increases.

## Discussion

The results support the hypothesis that humans adapt to virtual increases in motor noise by increasing antagonistic co-activation, and this was associated with reduced noise-induced performance decrements (Hypothesis 1). On the other hand, the results did not support the hypothesis that humans adapt to virtual decreases in motor noise by decreasing antagonistic co-activation, and decreasing noise did not improve performance (Hypothesis 2).

### Learning Trends in Non-Manipulated Trials

Subjects were able to improve their control of the virtual arm over the first day of practice, decreasing their average end-point error and movement time. While these quantities had excellent retention and plateaued after the first day, co-activation had poorer retention and continued to decrease throughout practice. A reduction in co-activation with practice is consistent with numerous prior studies (Thoroughman and Shadmehr, 1999; Osu et al., 2002; Gribble et al., 2003; Darainy and Ostry, 2008; Huang et al., 2012), which could reflect fine-tuning of an internal model of the virtual arm dynamics (Osu et al., 2002). However, few studies quantify *retention* of co-activation following skill acquisition. Patten and Kamen (2000) showed good retention of antagonistic co-activation levels for young and older adults performing a force-modulating task, but these data were averaged over a week of practice, which may occlude more transient changes in co-activation strategies. If antagonistic co-activation is indeed reflective of internal model tuning, the present results suggest that this fine-tuning was lost between practice sessions, and that learning the model was a relatively slow process, which could be because of the novelty associated with the virtual arm interface. However, since there are few reports on the retention of antagonistic co-activation strategies, further experimental work is needed to reach a firmer conclusion.

### Assessment of Motor Noise

Motor noise was operationally defined as high-frequency fluctuations in the linear envelopes of muscle activity recorded with surface electromyography. In contrast, past experimental studies have measured noise in terms of either the variability of motor neuron firing rates using indwelling electrodes (Clamann, 1969; Matthews, 1996), or the variability of force output during isometric tasks (Schmidt et al., 1979; Slifkin and Newell, 1999; Laidlaw et al., 2000). In the present study the scaling of motor noise, relative to the magnitude of the motor command, had a CV close to 0.2 for both muscles, which agrees with studies reporting CVs between 0.1 and 0.3 for the firing rate of motor neurons (Clamann, 1969; Matthews, 1996). These values are greater than CVs reported for isometric force production, which are typically less than 0.06 (Schmidt et al., 1979; Slifkin and Newell, 1999; Laidlaw et al., 2000). This is likely because the filtering properties of muscle and associated electromechanical processes (e.g., excitation-contraction coupling) attenuate higher-frequency fluctuations in the muscle force time-histories.

Of theoretical interest is how the CV scales with the magnitude of the control signal, i.e., the muscle activity linear envelopes. For example, the CV is assumed to be constant by optimal control models of human motor control (Harris and Wolpert, 1998). To determine this scaling, linear functions were fit to the log-log relation between the average and STD of the muscle activity linear envelopes for each subject (a constant CV gives a slope of 1.0). The average slope was 0.84, which is lower than that reported by Jones et al. (2002) for isometric finger force data, who found an average slope of 1.05. However, there was a wide range of variation in the data of Jones et al. (2002) i.e., if you remove the slope value for one outlier subject, the average slope becomes closer to the present study. Compared to the quality of the linear fits reported by Jones et al. (2002; reported as *R^2^* = 0.80 ± 0.1), the fits in the present study were stronger (*R^2^* = 0.94 ± 0.02 and 0.94 ± 0.04 for the biceps and triceps, respectively). This could be because the mechanical properties of muscle distort the relationship between neural activation and force, and therefore, assessing motor noise at the muscle activity level may be advantageous in this respect, but caution should still be exercised due to the greater potential for artifacts associated with electromyography.

### Effects of Increased Noise

The initial effect of increasing motor noise was to make performance worse without any change in antagonistic co-activation, but with additional practice subjects increased co-activation and were able to maintain their speed and accuracy in light of a virtual increase in motor noise. This is consistent with the hypothesis that co-activation suppresses task-level variability induced by signal-dependent motor noise (Hypothesis 1). The co-activation increase seems to reflect purposeful neural adaptation, as it goes against the steady decline of co-activation observed on non-manipulated trials, and is not an artifact of faster movements (Gribble and Ostry, 1998; Suzuki et al., 2001), because velocity did not change significantly during later time points. Most subjects used co-activation to stabilize the switch from accelerating the virtual arm towards the waypoint, to accelerating the virtual arm back towards the starting area (see muscle activation patterns displayed in Figure 2). This switch is a critical control point: if the deceleration is just right the virtual arm will smoothly come to a stop on the target (the frictional model assists slowing down), providing faster movement times than making corrections near the goal. Co-activation could help in regulating variability in the net joint torque during this switch (Gordon and Ghez, 1984; Ghez and Gordon, 1987; Selen et al., 2005). These results contrast with the those of de Rugy et al. (2012), who saw virtually no effect of a similar manipulation in a virtual force-directing task. This could be because in the experiment of de Rugy et al. (2012) musculotendon and rigid-body dynamics were excluded from the virtual arm model, which may have prevented subjects from taking advantage of the impedance-modulating capabilities of antagonistic muscles (Hogan, 1984).

### Effects of Decreased Noise

When noise was decreased movements became less accurate with no significant change in antagonistic co-activation. As with the noise increase, an initial decrement in performance would be expected after exposure to a novel task perturbation. However, unlike the noise increase, the decrease in accuracy persisted into the second manipulation block, and although co-activation appeared to reduce, the reduction was not significant. This may be because: (1) A longer period of adaptation may have been needed; (2) There may have been a floor effect, which made it difficult to further reduce co-activation; (3) The small, but unavoidable delay introduced by the filter may have made the task more difficult; (4) The filtering benefits may have been offset by a reduction in peak virtual muscle force. As can be seen in the exemplar data presented in Figure 2B, the normally sharp peaks in muscle activity are attenuated by the filter. Therefore, to get the same peak activation, subjects would need to activate their muscles more, which would increase signal-dependent noise, negating the benefits of noise-reduction. Note that subjects were still able to complete the task successfully with the filtered muscle activity, as their error only increased a few degrees and their movement time did not change significantly; and (5) In response to the noise reduction, the small (but non-significant) decrease in co-activation could have reduced the noise-suppression benefits of co-activation, again negating the positive effects of the noise reduction. The results of this experiment do not follow those of Chu et al. (2013) who found that dystonic children can take advantage of a perceived decrease in their motor variability in a throwing task. This may be because the present study investigated healthy adults, and may also be due to task differences (on-line control of a myoelectric virtual arm vs. a discrete throwing task).

### Implications

The data suggest that in the short-term, the nervous system adapts to increased signal dependent noise by increasing antagonistic co-activation. This could explain why older adults generally have increased levels of co-activation (Izquierdo et al., 1999; Macaluso et al., 2002), which could be due to a greater need to protect and stabilize joints (Huang et al., 2012) and/or the need to counter the effects of greater signal dependent noise. Greater motor noise in older adults has been documented by Laidlaw et al. (2000), who showed an increase in the CV of motor neuron firing rates at low force levels in a hand muscle, which was roughly double that of young adults. Similar findings have been observed in larger leg muscles, although to a lesser degree (Kallio et al., 2012). While greater signal dependent noise may require greater co-activation to compensate and maintain motor function, it is unknown whether the short-term adaptations observed in the present study would accurately reflect the much longer-term adaptations that would be expected over decades of aging. In addition, there is evidence that practice reduces motor unit discharge variability and increases force steadiness in older adults (Keen et al., 1994; Laidlaw et al., 1999; Kornatz et al., 2005), which could be due to changes in the low-frequency modulation of motor unit discharge rates (Dideriksen et al., 2012). Thus, in the long-haul, with training older adults could reduce their noise and thus, lessen the need for energetically expensive antagonistic co-activation.

### Limitations

Only short-term adaptations were studied, and therefore, it is unknown whether the results would hold if subjects continued to practice under the manipulated conditions. Also, the virtual arm was a relatively simple one-degree-of-freedom rigid body model actuated by a pair of antagonistic lumped muscle models. The muscle models were designed to mimic the behavior, but not the fine details, of human muscle. The advantage of this simple approach is that subjects only needed to learn to coordinate two muscles, and were able to become proficient at controlling the virtual arm in a relatively short time. However, in real human arms there are many more degrees of freedom, and it is unknown whether in this case a different solution could be found by the nervous system. Another limitation is that proprioceptive feedback related to the virtual arm was limited as the participants’ arms did not move during performance of the task (although there would still be feedback from muscle-tendon length changes and cutaneous receptors; see Figure 1). It is also unclear how the findings would generalize, as the noise manipulations were performed on healthy young adults. Further research is needed to see if other individuals, such as the elderly or patient populations, would respond in the same way. Finally, the observed changes in co-contraction cannot be separated into the contributions from various neural systems, such as movement commands vs. reflex activity (Gribble et al., 2003).

A potential criticism of the present approach is that subjects’ actual motor noise was unchanged, and ultimately, the manipulation was effectively a visual one. While it is true that subjects’ actual motor noise was not manipulated, adding variability to subjects’ muscle activity is not the same as adding variability to the visual display. The former preserves the causal ordering of events: variations in muscle activity produce variations in muscle force, which in turn affect fluctuations in net joint torque, and limb acceleration, velocity, and displacement. At each stage the fluctuations are filtered by various physiological processes (e.g., excitation-contraction coupling; muscle dynamics) and mechanics associated with these events (e.g., inertial properties of the arm). If the variability was introduced at a later stage, for example in the displayed motion of the virtual limb, the variations produced by the added noise would become causally disconnected from the subjects’ motor commands, decreasing the ecological validity of the task. Further study is needed to determine how the central nervous system responds to noise introduced at different points in the human motor control loop, whether in the motor command or in sensory feedback. The latter could be in the form of a visual perturbation, such as error amplification (Wei et al., 2005a,b), or a proprioceptive disruption, such as tendon vibration (Cordo et al., 1995, 2009).

## Conclusion

This work suggests that the nervous system adapts to virtual increases in motor noise by increasing antagonistic co-activation, which preserves motor performance. A virtual reduction in motor noise failed to benefit performance, but this may have been due to characteristics of the filtering process itself, e.g., delays are introduced and muscle activity bursts are attenuated. The observed adaptations to increased noise could explain in part why older adults and many patient populations have greater antagonistic co-activation, which may represent an adaptation to increased motor noise, along with a desire for increased joint stability. However, a downside is an increase in energy cost. Future work should determine whether the results hold in the long-term, or if the nervous system finds other ways to compensate for increases in motor noise.

## Author Contributions

CJH conceived and designed the experiments. CJH, OG, and GW performed the experiments. CJH analyzed the data and drafted the manuscript. CJH, OG, and GW read and approved the final manuscript.

## Conflict of Interest Statement

The authors declare that the research was conducted in the absence of any commercial or financial relationships that could be construed as a potential conflict of interest.
